# Resolution of Autoimmune Hepatitis Following Total Colectomy in Severe Ulcerative Colitis: A Rare Presentation Associated With Leaky Gut

**DOI:** 10.7759/cureus.19276

**Published:** 2021-11-05

**Authors:** Matthew A Heckroth, Mohamed Eisa, Matthew Cave

**Affiliations:** 1 Department of Internal Medicine, University of Louisville School of Medicine, Louisville, USA; 2 Department of Gastroenterology and Hepatology, University of Louisville School of Medicine, Louisville, USA

**Keywords:** inflammatory bowel disease, leaky gut, total colectomy, autoimmune hepatitis, ulcerative colitis (uc)

## Abstract

Liver disease is a common manifestation of inflammatory bowel disease. Although less common than other hepatic manifestations, autoimmune hepatitis can be seen in patients with ulcerative colitis. One possible mechanism in the relationship between the two is through the leaky gut where defects in the colonic mucosal barrier increase exposure to gut-derived toxins and bacterial products leading to immune activation. We report two cases of autoimmune hepatitis with concomitant severe ulcerative colitis in which complete remission of autoimmune hepatitis was seen following total colectomy.

## Introduction

Hepatobiliary dysfunction is a common extraintestinal manifestation of inflammatory bowel disease (IBD), especially ulcerative colitis (UC). Some of the common liver and biliary tract disorders associated with UC include primary sclerosing cholangitis (PSC), non-alcoholic fatty liver disease (NAFLD), cholelithiasis, as well as drug-induced liver injury secondary to medications used in the treatment of UC [1]. Although less common, another well-established complication of UC is autoimmune hepatitis (AIH) [2]. A proposed mechanism in the relationship between UC and AIH is through the leaky gut in which disruptions in colonic permeability allow for immune system activation in the liver [3]. We present two cases with concomitant severe UC and AIH, developing remission of their AIH after total colectomy.

## Case presentation

Case 1

A 60-year-old male initially presented in May 2014 with an asymptomatic elevation of transaminases (Table 1). His workup revealed positive antinuclear antibody (ANA) titer of 1:320 (negative <1:40) with a speckled immunofluorescence pattern and an elevated immunoglobulin G (IgG) of 3,190 mg/dL (normal range 751-1,560 mg/dL), consistent with AIH. Liver biopsy revealed necrosis and advanced stage 3 fibrosis and he was initiated on azathioprine and budesonide with improvement in liver function. Around the time of his AIH diagnosis, he developed hematochezia, which he initially did not disclose as it resolved with the initiation of steroids for his AIH. In February 2015, his rectal bleeding recurred and colonoscopy showed evidence of UC with pancolitis. UC symptoms remained uncontrolled until he ultimately underwent total colectomy with ileostomy in July 2017. The liver function returned to normal and azathioprine and budesonide have been discontinued for over four years, and AIH remained in remission.

**Table 1:** Liver function trend of patient 1. ALP: alkaline phosphatase; AST: aspartate aminotransferase; ALT: alanine aminotransferase.

	May 2014	March 2016	June 2017	April 2018	May 2020
Total bilirubin (mg/dL)	0.4	1.8	0.4	0.6	0.3
ALP (U/L)	100	139	167	43	40
AST (U/L)	145	42	26	27	19
ALT (U/L)	153	33	23	22	16
Albumin (g/dL)	2.9	2.1	3.1	4.3	3.8

Case 2

A 58-year-old female with a history of UC diagnosed three years prior was found to have elevated transaminases. She had positive smooth muscle antibodies at 75 U (negative <20 U), a positive ANA titer of 1:1,280 with a homogenous immunofluorescence pattern, and an elevated IgG of 2,333 mg/dL. Liver biopsy showed bridging fibrosis and subtle ductular reaction, which can be seen in PSC, with no evidence of AIH. According to the simplified criteria for diagnosis of AIH, proposed by the International Autoimmune Hepatitis Group, she had probable disease based on laboratory findings of positive smooth muscle antibodies, positive ANA titer, elevated IgG, and the absence of viral hepatitis. Liver biopsy, however, was more suggestive of PSC so she was initially diagnosed with AIH +/- small duct PSC. As the diagnosis of PSC is most frequently established using cholangiography, she had a magnetic resonance cholangiopancreatography (MRCP), which showed no focal strictures or saccular dilation of the bile ducts making PSC less likely. She was subsequently treated with azathioprine for AIH. She ultimately developed clinical cirrhosis as evidenced by thrombocytopenia, elevated international normalized ratio (INR), non-bleeding esophageal varices, and mild portal hypertensive gastropathy on esophagogastroduodenoscopy (EGD) 18 months following diagnosis of AIH. One year following her AIH diagnosis, she had a total colectomy with J-pouch. Intraoperative cholangiogram was again negative for PSC. Two years following colectomy, she had a repeat liver biopsy with a complete histopathological resolution of AIH cirrhosis as well as hepatic vein pressure gradient showing no portal hypertension and repeat EGD with no varices. She remains on 25 mg of azathioprine for interstitial lung disease (ILD) thought secondary to UC.

## Discussion

Disorders of the liver and biliary tract are common extraintestinal manifestations of IBD. The mechanisms are ongoing inflammation of the bowel or medications used for IBD (such as anti-tumor necrosis factors). Autoimmune hepatitis, unlike PSC and other causes of hepatobiliary disease, is not commonly seen in patients with UC. One proposed mechanism of the development of AIH in UC patients is via leaky gut [3], which has also been implicated in the pathogenesis of PSC.

The liver receives approximately 75% of its blood supply from the intestine through the portal vein and is exposed to many of these gut-derived products [3]. The intestinal epithelial lining forms a protective barrier separating the host from environmental pathogens. Patients with UC display multiple defects in this mucosal barrier resulting in increased intestinal permeability and exposure to many toxins, which lead to immune system activation. Also, bacterial translocation and endotoxins travel into the portal tract with subsequent activation of inflammatory cells and Kupffer cells, which leads to hepatocellular injury [4,5].

Most of the literature suggests that PSC progresses independently from UC. Hence colectomy for severe UC may not affect PSC activity. In fact, chronic antibiotic refractory pouchitis is commonly seen in patients with concomitant UC and PSC. These patients have a higher risk of cancer in the resected specimen as well as higher long-term mortality following proctocolectomy [4]. PSC/AIH is a known entity and half of the cases are usually in patients with IBD [6].

Autoimmune hepatitis in IBD is a less common occurrence, and the effect of UC on liver disease activity is not well known. Olsson and Hultén hypothesized that while PSC is typically found in patients with a more mild UC, AIH is typically seen in patients with more severe colitis [7]. They also found that disease activity of AIH was severely reduced following proctocolectomy as shown by disappearance or decreased titers of smooth muscle and antinuclear antibodies and immunoglobulins. This is in contrast to PSC in which perinuclear antineutrophil cytoplasmic antibody (pANCA) often persists following colectomy [7].

## Conclusions

Hepatobiliary dysfunction, especially PSC, is commonly seen in patients with ulcerative colitis. Autoimmune hepatitis is less commonly seen in these patients although an established relationship is known. Their association may be attributed to leaky gut in which disruptions in the protective colonic barrier allow for immune activation through the gut-liver axis. As we describe in our two patients, by removing the source via total colectomy, bacterial products and gut-derived toxins are no longer able to disrupt liver homeostasis and may improve AIH in patients with severe UC. Colectomy not only improved intestinal bacterial endotoxicity but also may have changed immune dysregulation. While we do not recommend colectomy to treat AIH in patients with severe UC, we suggest more research is needed in the gut-liver axis in AIH.
